# The Influence of Sports Participation on Body Image, Self-Efficacy, and Self-Esteem in College Students

**DOI:** 10.3389/fpsyg.2019.03039

**Published:** 2020-02-05

**Authors:** Yiyi Ouyang, Kun Wang, Tingran Zhang, Li Peng, Gan Song, Jiong Luo

**Affiliations:** ^1^Research Centre for Exercise Detoxification, College of Physical Education, Southwest University, Chongqing, China; ^2^Key Lab of Physical Fitness Evaluation and Motor Function Monitoring, Southwest University, Chongqing, China

**Keywords:** body image, self-efficacy, self-esteem, sports participation, mediating

## Abstract

**Objectives:**

This study aimed to explore the relationship between body image, self-efficacy, self-esteem, and sports participation by gender, grade, and specialty and then to provide a reference for promoting participation in sports and physical activities in college students.

**Methods:**

Using stratified random sampling, undergraduate students in western China were selected as participants. The data obtained in this study were processed by SPSS 19.0 and AMOS 21.0 statistical software.

**Results:**

Body image was significantly positively correlated with self-efficacy, self-esteem, and sports participation. Self-efficacy was significantly positively correlated with self-esteem and sports participation. Self-esteem was significantly positively correlated with sports participation. Body image had a direct effect on sports participation, with an effect value of 0.124. Furthermore, the mediating effects of self-efficacy (0.079) and self-esteem (0.108) were significant in the relationship between body image and sports participation. Meanwhile, the chain mediating role of self-efficacy–self-esteem was also obvious (0.035).

**Conclusion:**

Body image, self-efficacy, and self-esteem had significant influence on sports participation in college students. At the same time, the mediating effect of self-efficacy, self-esteem, and self-efficacy–self-esteem on body image and sports participation were established, and self-esteem was the key factor to sports participation.

## Introduction

Body image refers to the image formed by the individual to one’s own body, which is the objective cognition, and subjective assessment of one’s own body characteristics. It is composed of appearance, body shape, physical strength, health, and other dimensions, and the degree of self-awareness will affect emotion and health behavior, such as weight control, personal social adaptation, psychological stress, self-development, and interpersonal relationship (Wang et al., 2017). In today’s highly developed network media, people tend to focus on their own body shape and appearance but ignore health. Overexposure of good body shape will lead to low body satisfaction, thus increasing the risk of suffering from chronic disease and mental disorders and inhibiting exercise participation (Yu et al., 2012; Gillen, 2015; Andrew et al., 2016; Sun et al., 2017). Relevant research found (Ricciardelli and McCabe, 2004; Hu, 2017) that the level of personal satisfaction with body image has a strong correlation with the degree of sports participation. The more positive the body image is, the higher the degree of sports participation is; on the contrary, those who are dissatisfied with their body images or hold negative body images have an inhibitory effect on exercise behavior. As the generation of exercise behavior comes from exercise motivation and intention, the generation of both derives from people’s cognition, assessment, and attitude. The better body perception and movement perception can make college students fully show themselves (Bandura et al., 1980), so the students’ self-efficacy will have an impact on their exercise participation behavior. The higher the self-efficacy is, the higher the exercise participation degree is; otherwise, the lower the exercise participation degree is (Sun, 2010; Koring et al., 2012; Parschau et al., 2013).

Self-efficacy refers to the subjective prediction of one’s ability to accomplish a certain task, as well as the individual’s tendentious judgment and feeling on whether one’s behavior can achieve a certain goal (Guo et al., 2010; Ma and Yan, 2013; Li and Zhou, 2016). It has three implications: first, it belongs to the category of perception ability, but it is not equal to ability; second, the expectation of achieving a certain goal comes before the activity; third, it is a subjective judgment on whether one can achieve a certain goal. People with higher self-efficacy can complete the original sports participation plan (Sonstroem and Morgan, 1989; Zhang et al., 2010) when they confront difficulties; self-efficacy could not only stimulate the motivation level of individuals but also determine the level of individual participation in sports (Annesi and Mareno, 2015). In recent years, some scholars have found that (Ma, 2019) body image has a positive correlation with self-efficacy and that one with more positive body image has a better sense of self-efficacy. Meanwhile, Hausenblas et al. (2004) pointed out that body image has a positive predictive effect on self-efficacy and that body image and self-efficacy also have a positive impact on sports participation. Some scholars (Wu et al., 2006; Annesi and Mareno, 2015) also believed that female college students’ body image can play a role in physical exercise behavior through self-efficacy and that body image can also predict the level of self-efficacy directly and positively, which seems to suggest that self-efficacy may play a mediating role between body image and sports participation.

Some scholars (Liu et al., 2016; Cao and Zhang, 2018; Liao et al., 2019) also found that self-efficacy also has a positive impact on self-esteem and that we can promote individual self-esteem through improving self-efficacy. Self-esteem has a positive or negative attitude toward itself, which is holistic and specific. One may assess many of one’s own characteristics or integrate them to form a holistic evaluation. Therefore, self-esteem is a kind of emotional evaluation of individuals on themselves, and it is an important factor to predicting the implementation of health behavior. It has a positive effect on the degree of sports participation (Hülya et al., 2006). Self-esteem may also be affected by social values, the degree and evaluation of attention to things. In addition, college students pay too much attention to body shape, and the society over-publicizes the ideal body model, which led to the unbalanced state of one’s expectation on one’s body, resulting in negative emotions, and low self-esteem imagination (Borges et al., 2010; Beatrix et al., 2013). Peng et al. (2017) believed that self-esteem has a boosting effect on sports participation behavior, self-esteem is also one of the motivations to drive sports participation, and body image has a significant positive effect on self-esteem. To improve the degree of sports participation among college students, we should not only pay attention to how to establish the correct body image in contemporary college students but should also not ignore the improvement of their self-esteem. This seems to suggest that self-esteem may play a mediating role between body image and sports participation.

In summary, a positive body image among college students can enhance self-esteem and form a stable sense of self-esteem. It is a key factor to better implement the behavior of sports participation. Those with higher self-efficacy will have a positive impact on the level of individual self-esteem; a lower self-efficacy will lower the level of an individual’s self-esteem, while college students’ body image can positively predict self-esteem and can also indirectly influence self-esteem via self-efficacy. However, few scholars in previous studies have explored the relationship between body image, self-efficacy, self-esteem, and sports participation. Particularly, there is a lack of in-depth studies on the chain mediating mechanism of “self-efficacy–self-esteem.” Based on this, this study constructed a chain mediating model of body image, self-efficacy, self-esteem, and sports participation, and by taking body image, self-efficacy, and self-esteem as a personality system, the influence on sports participation has been examined. This study revealed the relevant mechanisms that affect participation in sports and better promote the development of autonomous habits in college students, thus providing a practical reference for enhancing their physical and mental health. The following hypotheses were put forward: (1) Self-efficacy is the mediator between body image and exercise participation; (2) self-esteem is the mediator between body image and exercise participation; and (3) self-efficacy and self-esteem play a chain mediating role between body image and exercise participation.

## Subjects and Methods

### Subjects

A total of 1,000 participants voluntarily participated in this study. This was sample randomized according to a stratified method by specialty and grade from a comprehensive university in Western China. With the help of physical education teachers and college psychological counselors, the survey was conducted anonymously after being approved by the ethics committee of Southwest University (approval no. 20190601C1). Of 1,000 questionnaires obtained, 887 were valid, with a return rate of 88.7%. For details, see Table 1.

**TABLE 1:** Basic information of the sample (*N* = 887).

**Variable**	**Mean ± SD**	**Variables**	**Category**	***n***	**%**
Age (years)	20.91 ± 1.39	Gender	Male	472	53.21
Height (cm)	172.19 ± 8.08		Female	415	46.79
Weight (kg)	65.88 ± 11.36	Grade	Freshman	234	26.38
BMI (kg/m^2^)	22.13 ± 2.92		Sophomore	211	23.79
			Junior	245	27.62
			Senior	197	20.74
		BMI	Underweight	217	24.46
			Normal	486	54.79
			Overweight	184	20.74

*BMI, body mass index = body weight (units in kg)/height (units in m^2^). Underweight BMI < 20 kg/m^2^, normal BMI: 20–25 kg/m^2^. Overweight BMI > 25 kg/m^2^ (above BMI grouping according to the Ministry of Education of China criteria for college students).*

### Methods

#### Questionnaire Design

With a structured questionnaire design method, the questionnaire consists of five components:

(1) The personal background information
  This component covers the basic background information on the subject’s gender, grade, height, weight, and body mass index (BMI), etc.
(2) The degree of sports participation scale
  With Fox’s (1999) three-question test method, the calculation of sports participation is as follows: sports participation = exercise frequency × (average exercise intensity + exercise duration). The range of score was 2–72 points, and the higher one scores, the higher is the degree of one’s sports participation. The exercise frequency is determined by exercise days each week, and the numbers 1–6 represent “0 day/week,” “1 day/week,” “2 days/week,” “3 days/week,” “4 days/week,” and “≥5 days/week,” respectively. The duration of exercise was determined by the average time participating in each exercise day; the numbers 1–6 represent “0–10 min/day,” “11–20 min/day,” “21–30 min/day,” “31–40 min/day,” “41–50 min/day,” and “≥51 min/day,” respectively; the average exercise intensity by the degree of fatigue of the subject after exercise, using the numbers 1–6 to represent “extremely relaxed,” “very relaxed,” “easy,” “a little tired,” “very tired,” and “extremely tired,” respectively.
(3) The self-esteem scale
  This scale was developed by Rosenberg and simplified by Tian (2006). Using 4 points to score from strongly disagree (1 point) to strongly agree (4 points); the second, fifth, sixth, eighth, and ninth questions in the scale were reversed; and the reverse questions were scored in reverse. The scale consists of two dimensions with a total of 10 items. Among them, dimension 1 (self-affirmation) contains five items; the higher the score is, the more positive the self-affirmation is. Dimension 2 (self-denial) also contains five items; the higher the score is, the more negative the self-affirmation is.
(4) The body image scale
  The scale was developed by Cash et al. (1990). It contains five dimensions, namely, physical fitness evaluation, appearance adaptation, weight concern, appearance evaluation, and health evaluation. The scale was scored using the 5-point Likert scale. The options were “strongly agree, agree, neither disagree nor agree, disagree, and strongly disagree,” which were scored as 5, 4, 3, 2, and 1, respectively, and the higher the score, the more positive the body image. Among the five dimensions, for the physical fitness evaluation (11 items), the higher the score, the higher the conscious ability to have better physical and athletic ability; for the weight concern (four items), the higher the score, the more attention to body weight; for the appearance adaptation (four items), the higher the score, the more attention to one’s appearance; for the appearance evaluation (six items), the higher the score, the more positive and satisfactory the appearance; for the health evaluation (four items), the higher the score, the better the health condition, and physical fitness.
(5) The self-efficacy scale
  This scale has been developed by Wang et al. (2001), consisting of 10 questions. Each question had four options: “completely incorrect,” “somewhat correct,” “fairly correct,” and “completely correct.” The corresponding scores were 1–4 points, and the higher the total score, the stronger the self-efficacy.

#### Reliability and Validity of the Questionnaire

After four rounds of expert consultation, the expert scoring method was adopted, that is, whether the design of each question reflects the research subject to be explored and experts being asked to make an evaluation. Exploratory factor analysis and structural equation model were used to explore the potential structure of variables (Table 2). The structural validity and reliability of the three scales were explored separately.

**TABLE 2:** Reliability and structural validity statistics of the three scales.

**Scale**	**Kaiser–Meyer–**	**Factor**	**Entry**	**Explanation**	**Progressive**	**Combination**	**Cronbach**
	**Olkin (KMO) and**	**naming**	**numbers**	**variance %**	**interpretation**	**reliability**	**α coefficient**
	**Bartlett l test**				**variance %**		
The self-esteem	KMO = 0.85	Self-affirmation	5	3.681	36.809	36.809	0.78
scale^1^	*P* < 0.000	Self-denial	5	1.530	15.296	52.105	0.74
The body image	KMO = 0.91	Physical fitness evaluation	11	8.965	30.913	30.913	0.89
scale^2^	*P* < 0.000	Appearance adaption	4	2.563	8.839	39.752	0.85
		Weight concern	4	2.172	7.489	47.241	0.79
		Appearance evaluation	6	1.674	5.771	53.013	0.86
		Health evaluation	4	1.401	4.833	57.845	0.79
The self-efficacy	KMO = 0.85	Self-efficacy	6	3.193	53.214	53.214	0.80
scale^3^	*P* < 0.000						

*^1^The verification results of the self-esteem scale: *X*^2^/DF = 1.639, adjusted goodness-of-fit index (AGFI) = 0.943, comparative fit index (CFI) = 0.991, Tucker–Lewis index (TLI) = 0.980, incremental fit index (IFI) = 0.991, goodness-of-fit index (GFI) = 0.979, and root mean square error of approximation (RMSEA) = 0.051. Cronbach α coefficient is 0.80. ^2^The verification results of the body image scale: *X*^2^/DF = 1.539, AGFI = 0.945, CFI = 0.981, TLI = 0.987, IFI = 0.971, GFI = 0.976, and RMSEA = 0.049. Cronbach α coefficient is 0.91. ^3^The verification results of the self-efficacy scale: *X*^2^/DF = 1.609, AGFI = 0.931, CFI = 0.928, TLI = 0.962, IFI = 0.954, GFI = 0.946, and RMSEA = 0.036. Cronbach α coefficient is 0.82.*

It is not difficult to find the following from Table 2:

(1) The Kaiser–Meyer–Olkin (KMO) values of the three subscales were 0.85, 0.91, and 0.85, respectively, and the Bartlett spherical test values reached significant levels, indicating that the three subscales were suitable for factor analysis.
(2) Two common factors can be extracted from the self-esteem scale, and its cumulative contribution rate can explain 52.11% of the total variation. The internal consistency Cronbach alpha coefficients were 0.78, 0.74, and 0.80, respectively. The verification results of the measurement model were *X*^2^/DF = 1.639, and the values of the adjusted goodness-of-fit index (AGFI), comparative fit index (CFI), Tucker–Lewis index (TLI), incremental fit index (IFI), goodness-of-fit index (GFI), and root mean square error of approximation (RMSEA) were 0.943, 0.991, 0.980, 0.991, 0.979, and 0.051, respectively. The data indicated that the measurement model fitted well with the survey data. It showed that the scale structure had good validity.
(3) Five common factors can be extracted from the body image scale, and its cumulative contribution rate can explain 57.85% of the total variation. The internal consistency Cronbach alpha coefficients were 0.89, 0.85, 0.79, 0.86, 0.79, and 0.91, respectively. The verification results of measurement model showed that *X*^2^/DF = 1.539, and the values of AGFI, CFI, TLI, IFI, GFI, and RMSEA were 0.945, 0.981, 0.987, 0.971, 0.976, and 0.049, respectively. The data indicated that the model and the survey data fitted well. It showed that the scale structure had good validity.
(4) One common factor was selected from the self-efficacy scale, and the cumulative contribution rate could explain 53.21% of the total variance. The internal consistency Cronbach alpha coefficient was 0.82. The verification results of the measurement modeled showed that the values of X^2^/DF = 1.609 and that AGFI, CFI, TLI, IFI, GFI, and RMSEA were 0.931, 0.928, 0.962, 0.954, 0.946, and 0.036, respectively. The data indicated that the model and the data obtained by the survey fitted well, which revealed that the scale structure was better.

### Statistical Analysis

Data obtained in this study were analyzed using SPSS 19.0 and AMOS 21.0 software packages. The normal distribution of all variables was tested using the Kolmogorov–Smirnov test, and all continuous variables follow the normal distribution. The statistical methods included descriptive statistics, independent-sample *t*-test, one-way ANOVA, exploratory factor analysis, correlation analysis, structural equation model, and bootstrap analysis, etc. The significance level of all variables was set as α = 0.05.

## Results

### Comparison of Differences in Body Image by Personal Background in College Students

Table 3 shows that (1) there were significant differences in physical fitness evaluation, weight concern, appearance adaptation, and appearance evaluation by gender, and health assessment (*t* = 0.39, *P* > 0.05) seemed to be unaffected by gender. It indicated that the scores of the males in physical fitness evaluation (*t* = 7.81, *P* < 0.001) and appearance evaluation (*t* = 6.10, *P* < 0.001) were significantly higher than those of the females, while the females were significantly higher in weight concern (*t* = −7.43, *P* < 0.001) and appearance adaption (*t* = −3.87, *P* < 0.001) than the males; (2) significant differences were observed in physical fitness evaluation (*F* = 16.21, *P* < 0.001), weight concern (*F* = 3.35, *P* < 0.05), appearance adaptation (*F* = 2.83, *P* < 0.05), appearance evaluation (*F* = 5.09, *P* < 0.05), and health evaluation by grade. It indicated that the scores of sophomores in physical fitness evaluation were significantly lower than those of freshmen, juniors, and seniors, while the scores of seniors were significantly higher in physical fitness evaluation than those of freshmen and sophomores; the scores of juniors in weight were significantly lower than those of freshman, sophomore, and senior students; the scores of freshmen were significantly lower in appearance evaluation than those of juniors and seniors; the scores of seniors were significantly higher in appearance evaluation than those of freshmen and sophomores; the scores of sophomores in health evaluation were significantly lower than those of freshmen, juniors, and seniors; and (3) significant differences in physical fitness evaluation (*F* = 12.87, *P* < 0.001), weight concern (*F* = 9.17, *P* < 0.001), appearance evaluation (*F* = 3.13, *P* < 0.05), and health evaluation (*F* = 4.47, *P* < 0.05) existed by BMI, while appearance adaptation (*F* = 2.32, *P* > 0.05) was not affected by BMI. It indicated that those with normal weight scored significantly higher in physical fitness than those with underweight; those with normal weight and overweight scored significantly higher on weight concern than those with underweight, while those with normal weight scored significantly higher in appearance evaluation than those with overweight; those with overweight had a significantly lower score in health evaluation than those with normal weight and underweight.

**TABLE 3:** Comparison of differences in body image characteristics in college students.

	**Physical fitness**	**Appearance adaption**	**Weight concern**	**Appearance evaluation assessment**	**Health evaluation**
**Gender**					
Male	31.67 (9.78)	10.19 (3.99)	10.37 (3.83)	14.66 (5.57)	6.35 (2.16)
Female	26.45 (9.31)	11.28 (4.10)	11.54 (4.06)	12.47 (4.67)	6.30 (1.99)
*t*-value	7.81***	−3.87***	−7.43***	6.10***	0.39
**Grade**					
Freshman	29.34 (9.82)	10.35 (4.05)	10.08 (3.89)	13.29 (5.36)	6.72 (2.12)
Sophomore	27.07 (8.67)	10.60 (4.15)	9.90 (3.91)	13.24 (4.66)	6.01 (2.04)
Junior	30.79 (10.54)	11.42 (3.53)	9.02 (4.29)	14.24 (5.30)	6.67 (2.06)
Senior	34.59 (11.23)	11.43 (4.30)	10.58 (3.75)	15.40 (6.52)	6.49 (1.99)
*F*−value	16.21***	2.83*	3.35*	5.09*	4.61*
LSD	1 > 2; 1 < 4; 2 < 3; 2 < 4	1 < 3; 1 < 4	1 > 3; 2 > 3; 3 < 4	1 < 4; 2 < 4	1 > 2; 2 < 3; 2 < 4
**BMI**					
Underweight	27.39 (9.37)	10.79 (4.03)	8.59 (3.96)	13.55 (4.75)	6.29 (2.03)
Normal	30.94 (10.37)	10.80 (4.18)	10.80 (3.91)	13.92 (5.77)	6.47 (2.08)
Overweight	30.15 (9.92)	9.56 (3.68)	12.78 (4.03)	12.03 (4.90)	5.57 (2.24)
*F* value	12.87***	2.32	9.17***	3.13*	4.47*
LSD	1 < 2		1 < 2; 1 < 3; 2 < 3	2 > 3	1 > 3; 2 > 3

*P < 0.05 and ***P < 0.001. *t*-value, the value of the independent-sample *t*-test; *F*-value, the value of the one-way ANOVA test; LSD, least square difference.

### Comparison of Differences in Self-Esteem and Sports Participation by Personal Background in College Students

Table 4 shows that (1) there was a significant difference in self-affirmation (*t* = 2.13, *P* < 0.05), self-efficacy (*t* = 6.69, *P* < 0.001), and sports participation (*t* = 5.03, *P* < 0.001) by gender, while self-denial (*t* = 0.02), *P* > 0.05) was not affected by gender. It showed that the scores of males were higher than those of females in self-affirmation, self-efficacy, and sports participation; (2) there were significant differences in sports participation (*F* = 13.29, *P* < 0.001) and self-negation (*F* = 4.45, *P* < 0.01) by grade, while self-affirmation (*F* = 1.20, *P* > 0.05) and self-efficacy (*F* = 1.33, *P* > 0.05) had no significant difference by grade. It showed that the scores of freshmen was significantly higher than those of sophomores, juniors, and seniors in self-denial; the scores of seniors were significantly higher in sports participation than those of freshmen and sophomores; and (3) self-efficacy (*F* = 13.89, *P* < 0.001) and sports participation (*F* = 11.47, *P* < 0.001) were significantly affected by BMI, but self-affirmation (*F* = 0.58, *P* > 0.05), self-denial (*F* = 1.09, *P* > 0.05) seem to have nothing to do with the BMI. Those with normal weight were significantly higher in self-efficacy and sports participation than those with underweight and overweight.

**TABLE 4:** Comparison of differences in self-esteem, self-efficacy, and sports participation characteristics in college students.

**Variables**	**Self-affirmation**	**Self-denial**	**Self-efficacy**	**Sports participation**
**Gender**				
Male	14.31 (2.82)	15.97 (2.98)	15.29 (5.39)	19.63 (11.22)
Female	13.88 (2.97)	16.63 (2.69)	12.68 (5.71)	16.04 (8.85)
*t*-value	2.13*	−0.02	6.69***	5.03***
**Grade**				
Freshman	14.15 (2.95)	17.06 (2.73)	13.88 (5.68)	16.75 (9.28)
Sophomore	13.91 (2.97)	16.24 (2.90)	13.80 (5.46)	16.80 (9.20)
Junior	14.47 (2.69)	16.59 (2.73)	13.38 (5.87)	20.10 (12.45)
Senior	14.29 (2.60)	16.73 (2.98)	14.93 (6.29)	23.34 (12.53)
*F*−value	1.20	4.45**	1.33	13.29***
LSD		1 > 2; 1 > 3; 1 > 4		1 < 4; 2 < 4
**BMI**				
Underweight	13.99 (2.85)	16.66 (2.55)	14.61 (5.62)	19.70 (11.23)
Normal	14.22 (2.96)	16.67 (3.03)	16.36 (5.48)	20.26 (10.34)
Overweight	14.13 (2.80)	16.07 (3.53)	12.93 (5.60)	15.93 (8.98)
*F*-value	0.58	1.09	13.89***	11.47***
LSD			1 < 2; 1 > 3; 2 > 3	1 < 2; 1 > 3; 2 > 3

*P < 0.05, **P < 0.01, and ***P < 0.001. *t*-value, the value of the independent-sample *t*-test; *F*-value, the value of the one-way ANOVA test; LSD, least square difference.

### Correlation Analysis of Body Image, Self-Efficacy, Self-Esteem, and Sports Participation in College Students

Pearson correlation was used to analyze the correlation coefficient between body image, self-esteem, self-efficacy, and sports participation (Table 5). The results showed that physical fitness evaluation, appearance adaptation, appearance evaluation, weight concern, and health evaluation in body image had significant positive correlation effects with self-affirmation and self-denial in self-esteem structure; physical fitness evaluation, appearance adaptation, appearance evaluation, weight concern, and health evaluation in body image had positive correlation effects with self-affirmation, self-denial, and self-efficacy in self-esteem structure, meanwhile, physical fitness evaluation, appearance adaptation, appearance evaluation, weight concern, and health evaluation in body image had positive correlation effects with self-affirmation, self-denial, and sports participation in self-esteem structure as well. The correlation between the variables was significant, which can provide a good basis for the subsequent exploration of the mediation effect test.

**TABLE 5:** Correlation analysis of body image, self-efficacy, self-esteem, and sports participation in college students (*N* = 820).

**Index**	**Mean ± SD**	**PFE**	**AA**	**AE**	**WC**	**HE**	**SA**	**SDE**	**SE**	**SP**
PFE	29.20 ± 9.90	1.00								
AA	10.71 ± 4.08	0.57***	1.00							
AE	13.62 ± 5.27	0.53***	0.48***	1.00						
WC	10.62 ± 3.95	0.52***	0.47***	0.54***	1.00					
HE	13.31 ± 4.01	0.43***	0.49***	0.46***	0.54***	1.00				
SA	13.11 ± 2.89	0.23***	0.25***	0.33***	0.19***	0.25***	1.00			
SDE	15.63 ± 2.84	0.22***	0.21***	0.24***	0.23***	0.17***	0.55***	1.00		
SE	24.91 ± 5.68	0.27***	0.24***	0.22***	0.29***	0.28***	0.31***	0.30***	1.00	
SP	17.94 ± 10.28	0.35***	0.22***	0.31***	0.27***	0.24***	0.35***	0.33***	0.39***	1.00

***P < 0.001. PFE, physical fitness evaluation; AA, appearance adaption; AE, appearance evaluation; WC, weight concern; HE, health evaluation; SA, self-affirmation; SDE, self-denial; SE, self-efficacy; SP, sports participation.

### Causal Relationship of Body Image, Self-Efficacy, Self-Esteem, and Sports Participation in College Students

**FIGURE 1:**
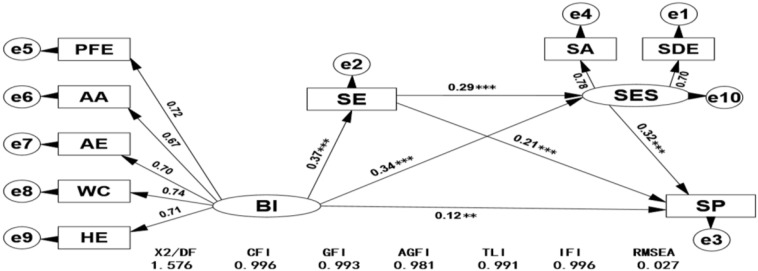
Diagram of path analysis of body image, self-efficacy, self-esteem, and sports participation as well as its model fitting test. PFE, physical fitness evaluation; AA, appearance adaption; AE, appearance evaluation; WC, weight concern; HE, health evaluation; SA, self-affirmation; SDE, self-denial; SE, self-efficacy; SP, sports participation; BI, body image; SES, self-esteem. ^∗∗^*P* < 0.01 and ^∗∗∗^*P* < 0.001.

In order to examine the relationship of body image with self-esteem, self-efficacy, and sports participation and to test the mediating role of self-esteem and self-efficacy, according to the mediating effect test procedure of Wen and Ye (2014), this study analyzes the relationship of body image, self-efficacy, and sports participation using the structural equation model by AMOS software. The model was modified to obtain the final model (Figure 1). The model fitting indexes were X^2^/DF = 1.576 < 2.000, CFI = 0.996, GFI = 0.993, AGFI = 0.981, TLI = 0.991, IFI = 0.996, and RMSEA = 0.027, which indicated that the model can be established well.

From the standardized path coefficient β values and significant levels in the mixed model structure in Figure 1, it is not difficult to find that there is a significant positive predictive effect from body image to self-efficacy (β = 0.37^∗∗^, *P* < 0.001), self-esteem (β = 0.34^∗∗^, *P* < 0.001), or sports participation (β = 0.12^∗∗^, *P* < 0.01). Self-efficacy (β = 0.32^∗∗^, *P* < 0.001) and self-esteem (β = 0.21^∗∗^, *P* < 0.001) both can significantly predict sports participation. In order to verify the mediating role of self-efficacy and self-esteem between body image and sports participation, the non-parametric percentile bootstrap program was used to test the mediating significance level. The sampling was repeated 5,000 times from the original data to calculate a 95% confidence interval (CI). If the standardized path coefficient 95% CI does not contain 0, the mediation was significant. From the body image through self-efficacy, self-esteem to sports participation, the chain of mediating role of 95% CI was (0.021, 0.061); from body image through self-efficacy to sports participation, the mediating role of 95% CI was (0.057, 0.115); and from body image through self-esteem to sports participation, the mediating role of 95% CI was (0.083, 0.158). The above three intervals did not contain 0, which indicated that the mediating effects were significant. Through further decomposition of the effect of each variable on sports participation, as shown in Table 6, the direct effect of body image to sports participation was 0.124, and the total mediating effect value of 0.222 was the sum of the mediating effects of the three mediating paths, that is, the total indirect effect. The sum of the direct effect and the total mediating effect was the total effect, that is, 0.346; the mediating effect value was divided by the total effect as the effect amount. The effect sizes of the three mediating paths in this study were 10.12%, 22.83%, and 31.21%, respectively.

**TABLE 6:** Decomposition table of path and its effects of body image acting on sports participation.

**Effect**	**Path relationship**	**Effect**	**Bootstrap SE**	**Bootstrap 95% CI**	**Relative mediating effect (%)**
Direction effect	Body image → sports participation	0.124	0.011	(0.109,0.152)	35.84
Indirection effect	Body image → self-esteem → self-efficacy	0.035	0.009	(0.021,0.061)	10.12
	→ sports participation				
	Body image → self-efficacy → sports participation	0.079	0.014	(0.057,0.115)	22.83
	Body image → self-esteem → sports participation	0.108	0.016	(0.083,0.158)	31.21
Total mediating effect		0.222	0.027	(0.178,0.286)	64.16

*SE, standard error; CI, confidence interval.*

## Discussion

### Impact of Personal Background on Body Image in College Students

This study found that female college students pay more attention to weight control and appearance adaptation, while male students care more about physical fitness and appearance evaluation, and it indicated that the females focused on their body and appearance more than the males, which was consistent with Zhang and Wang (2013) and other scholars, and it also was confirmed by Renée’s (2003) research. That is, the males emphasized masculine spirit, thereby by strengthening muscle and body training to present a physical image. In this study, it was found that the appearance adaptation score of freshmen was significantly lower than that of the senior.

This study found that freshmen were weak in appearance adaptation, sophomores scored low in physical fitness and health, junior students paid low attention to weight, and senior students were higher than other grades in physical fitness evaluation, appearance adaptation, weight attention, and appearance evaluation. This seemed to imply that freshmen were not as attentive as higher-grade students in their appearance. Sophomores may be the most arduous stage in their academic tasks; therefore, lack of exercise investment may lead to obvious disadvantages in physical fitness evaluation and health evaluation, while owing to the pressure of employment, the seniors may be forced to pay attention to body weight and body image again, so they scored the highest on appearance evaluation and weight concern. In addition, the study also found that those with normal BMI values were significantly higher in physical fitness evaluation and weight concern than those with underweight. However, there was no difference in appearance evaluation, but it was significantly higher than those with overweight. This showed that people with normal weight had better physical status than those with underweight, and they were more concerned about their weight status and appearance than those with overweight. This finding was consistent with Guo et al. (2017) research. That is, those who were overweight in health evaluation were lower than those who were normal weight and underweight; it indicated that those who were overweight may not be as good in health as the other two.

### Relationship of Personal Background With Self-Efficacy, Self-Esteem, and Sports Participation in College Students

The study found that males were more likely to have self-affirmation, self-efficacy, and sports participation than females. The reason for this may be that the males were better than the females in physical and motor skills, so they often participated in sports. At the same time, they can fully demonstrate themselves in their joy of sports and thus enhance their self-affirmation. This study found that freshmen had stronger self-denial, while seniors had a higher degree of exercise participation, and it also found that other grade students had less self-denial than freshmen. The reason may be that the freshmen’s incompatibility with the university model and their lack of maturity in their cognitive ability make them more likely to be in self-denial relative to other grades. The lower grades may be because the seniors had a lighter academic task and had more time for leisure, and the pressure of employment forced them to participate in sports to regulate their body–mind status. The study also found that BMI was significantly different in self-efficacy and sports participation. Students with normal weight were more active in self-efficacy and sports participation than other two groups; it indicated that those with normal weight were more attentive in self-efficacy and sports participation than those with underweight and overweight.

### Structured Path Model of Body Image, Self-Efficacy, Self-Esteem, and Sports Participation

This study found that body image has a remarkably positive effect on sports participation. This result showed that the higher the body image one has, the higher sports participation one has, which coincides with the research by Hu (2017). Body image significantly correlates with sports participation, and body image boosts sports participation. The research by Ricciardelli and McCabe (2004) has also found that body image exerts a positive effect on sports participation; namely, the more positive the body image, the more active the sports participation. According to relevant researches (Yu et al., 2012), college students are easily exposed to social and cultural pressure, which has negative influences on body satisfaction. Such influences come from families, peers, and the media, like the excessive attention on slenderness in family culture, peers’ laughing at overweight or obesity, and the media’s overexposure of good figures. All of these factors contribute to low body satisfaction, while the influence from the media plays a relatively large part. However, as most exercise behaviors involve body exposure in public places, those with poor body images often have increasing social physique anxiety, which hinders or reduces their sports participation; otherwise, those with good body images have high sports participation (Hu, 2017). Therefore, body image has a positive predictive effect on sports participation.

This study found that body image and self-efficacy had a significant positive influence on sports participation and that the higher the body image scores, the higher the degree of sports participation, which was consistent with the findings of the study by Hu (2017). While the higher the level of self-efficacy, the higher the level of sports participation, this result was consistent with the perspectives of Sun (2010), Koring et al. (2012) and Parschau et al. (2013). The study also found that body image had a significant positive effect on self-efficacy, and this result was consistent with Ma’s (2019) study; that is, body image was positively related to self-efficacy, and the more positive the body image, the more dominant the self-efficacy. Wu et al. (2006) and Annesi and Mareno (2015), and other scholars also believed that the body image of female college students can play a role in physical exercise behavior through self-efficacy, and body image can also directly predict the level of self-efficacy. In summary, combined with the bootstrap mediating test procedure, self-efficacy should act as an intermediary in body image and sports participation. It showed that college students can directly influence their sports participation by promoting a correct and positive body image and indirectly affect their sports participation behavior by improving self-efficacy. The first hypothesis that self-efficacy is the mediator between body image and exercise participation was established.

This study found that self-esteem had a significant positive effect on sports participation; a higher self-esteem led to a tendency to engage in and be attached to sports, and a lower self-esteem resulted in denial and alienation of sports. This result was nearly consistent with the findings of Korkmaz (2014) and You et al. (2017), and it has also proven Birkimer et al. (1996) findings that that self-esteem was helpful in promoting the sports participation, and self-esteem was also one of the motivations to drive sports participation. In addition, the study also found that body image had a significant positive effect on self-esteem; the more positive the body image, the higher the level of self-esteem, which was consistent with Guo et al. (2017) study. It has also confirmed that body image had a significant positive effect on self-esteem (Beatrix et al., 2013; Wang et al., 2016). In short, positive body image in college students can enhance self-esteem, and stable self-esteem can play an important role in promoting their participation in sports. Therefore, it can be inferred that self-esteem may play a mediating role between body image and sports participation behavior in college students. Therefore, it can be inferred that the second hypothesis that self-esteem may play a mediating role in the relationship between college students’ body image and exercise participation behavior is true.

This study found that the body image of college students had an impact on the degree of sports participation by self-efficacy, and self-efficacy affected the sports participation by partial mediation of self-esteem. This forms the mediating role chain between self-efficacy, self-esteem in body image, and sports participation. It showed the phenomenon that the lower the body image, the lower the self-efficacy, which led to the decline of self-esteem, and even self-denial and further hindered the level of participation in sports in college students. On the contrary, in college students, those with higher body image can promote self-efficacy, thus enhancing their sense of self-esteem and self-affirmation and promoting sports participation. Previous studies (Liu et al., 2016; Cao and Zhang, 2018; Liao et al., 2019) have affirmed the correlation between self-efficacy and self-esteem; that is, those with higher self-efficacy will produce positive effects, whereas those with lower self-efficacy will lower the level of self-esteem. As discussed above, body image can directly influence sports participation and can also indirectly influence the degree of sports participation by the indirect path of self-efficacy, mediating role of self-esteem, and the chain mediating role of “self-efficacy and self-esteem,” which provides an important practice reference to promote sports participation in college students. At the same time, it also supported the third hypothesis.

The shortcomings of this study were as follows: First, this study is cross-sectional, so we hope that future research can consider using the method of a follow-up study to further support the results of this study. Second, this study applies a holistic approach to explore the relationships among body image, self-efficacy, self-esteem, and sports participation. It is hoped that the successors can further explore and prove their relationships by gender, grade, BMI, and other background information. Finally, the research subjects of this study were composed entirely of university students in Western China; thus, future research could select a wider group of subjects to test the external validity of the results of this study.

## Conclusion

Body image in college students is affected by gender and BMI. It was shown that females pay more attention to weight concern and appearance adaptation, while males pay more attention to physical evaluation and appearance evaluation. Those with normal BMI had better physical evaluation and weight concern than those with underweight BMI. The self-affirmation, self-efficacy, and sports participation scores were significantly higher in the males than in the females, while self-efficacy and sports participation scores were significantly higher in those with normal BMI than in those with underweight and overweight. Self-efficacy and self-esteem were established as mediators of body image and sports participation, and the combination of self-efficacy and self-esteem was also established as a chain intermediary role of body image and sports participation.

## Data Availability Statement

All datasets generated for this study are included in the article/supplementary material.

## Ethics Statement

The studies involving human participants were reviewed and approved by the Hospital Ethics Committee of Southwest University in China. The patients/participants provided their written informed consent to participate in this study.

## Author Contributions

All authors designed this study, and contributed to and approved the final manuscript. YO and JL carried out the protocol and questionnaire survey. KW recruited the individuals with drug addicts. TZ and LP undertook the statistical analysis and graphical representation of the data. GS revised the draft.

## Conflict of Interest

The authors declare that the research was conducted in the absence of any commercial or financial relationships that could be construed as a potential conflict of interest.
